# How does Arab genome different from other genomes?

**DOI:** 10.1186/1471-2164-15-S2-O8

**Published:** 2014-04-02

**Authors:** Ibrahim Abdulkareem

**Affiliations:** 1 Molecular Pathology and Genetics, DPLM, King Abdullah International Medical Research Center, Kingdom of Saudi Arabia; 2 COM, King Saud bin Abdulaziz University for Health Sciences, King Abdulaziz Medical City, National Guard Health Affairs, Kingdom of Saudi Arabia

## 

Whole-genome sequencing results from Caucasian, African, Chinese and Korean individuals have been studied and published to date. Here, we successfully generated, assembled and analyzed the first full genome of an Arabic individuals (32 healthy volunteers from Saudi Arabia) using Next Generation Sequencing. Alignment of the Arabic genome with H19 references revealed nearly 450,182 unique single-nucleotide polymorphisms (SNPs) out of 3.18 million total SNPs, including 10,609 non-synonymous SNPs and 174,579 Deletion or Insertion Polymorphisms (DIPs). A de novo assembly of 9,011 contigs sequences was identified and which are not represented within the current human reference genome sequence maintained at NCBI. In addition, the assembled Arab mitochondrial genome was closest to the L0a haplogroup sequence of the Datog ethnic group of Tanzania.

